# SARS-CoV-2 Infection with Different Radiological Insights

**DOI:** 10.3390/diagnostics10050283

**Published:** 2020-05-07

**Authors:** Barbara Brogna, Claudia Brogna, Alberigo Martino, Stefana Minichiello, Domenico M. Romeo, Paolo Romano, Elio Bignardi, Emerico Maria Mazza, Lanfranco Musto

**Affiliations:** 1 Radiology Unit, “Frangipane” Hospital, ASL Avellino, Via V. Emanuele, Ariano irpino, 83031 Avellino, Italy; albmart@libero.it (A.M.); icomazza@alice.it (E.M.M.); musto.lanfranco@gmail.com (L.M.); 2 Neuropsychiatric Unit ASL Avellino, Via Degli Imbimbo 10/12, 83100 Avellino, Italy; claudiabrogna@yahoo.it; 3 Cardiologic Unit “Frangipane” Hospital, ASL Avellino, Via V. Emanuele, Ariano irpino, 83031 Avellino, Italy; stefana.minichiello@libero.it; 4 Neuropsychiatric Unit, Catholic University of Sacred Heart, Fondazione Policlinico Agostino Gemelli IRCCS, 00168 Rome, Italy; domenico.romeo@policlinicogemelli.it; 5 Radiology Unit, “Criscuoli” Hospital, ASL Avellino, Via Quadrivio, Sant’Angelo Dei Lombardi, 83054 Avellino, Italy; paolorom67@gmail.com; 6 Radiology Unit, “Cotugno Hospital, Naples, Via Quagliariello 54, 80131 Naples, Italy; dr.eliobignardi@alice.it

**Keywords:** severe acute respiratory syndrome-Coronavirus-2, lung acute embolism, acute respiratory distress syndrome

## Abstract

Severe acute respiratory syndrome-Coronavirus-2 (SARS-CoV-2) is a novel viral infection characterized by several symptoms range from mild to severe clinical conditions that could lead to death. We report two different radiological findings on computed tomography (CT) in two patients affected by SARS-CoV-2: a lung acute embolism (APE) in the first case and a radiological picture of acute respiratory distress syndrome (ARDS) in the second case. This is an important issue to be identified in order to provide more specific therapy earlier, including both antiviral and anti-inflammatory drugs associated with anti anticoagulant therapy.

## 1. Introduction

Coronavirus disease-19 (COVID-19) is a novel viral pandemic disease first detected in Wuhan, China, caused by severe acute respiratory syndrome Coronavirus-2 (SARS-CoV-2), with increasing incidence in the whole world and a wide spectrum of disease severity [1]. On 31 January 2020, the World Health Organization (WHO) declared a Public Health Emergency of International Concern due to the growing outbreak of COVID-19 in China. Despite travel restrictions, border control, and quarantine measures applied in over the world, many countries have experienced a rapid virus spread. Several reports in China, Italy, Spain, and the USA confirmed high mortality due to acute respiratory failure or other related complications.

SARS-CoV-2 belongs to the β-coronavirus, containing a single-stranded positive-sense RNA that encodes for both structural and non-structural proteins, including spike proteins that play a major role in virus entry and virus replication in the host cell via the receptor angiotensin-converting enzyme 2 (ACE2) [2]. The ACE2 protein has been identified in various human organs, including the respiratory system, GI tract, lymph nodes, thymus, bone marrow, spleen, liver, kidney cells, brain, and endothelia [3]. Although several studies confirmed that the SARS-CoV-2 shares 92% homology with the bat coronavirus sequence RaTG3, suggesting a zoonotic origin animal reservoir, enzootic patterns of transmission remain still uncertain [4]. However, possible recombination within the viral spike glycoprotein between the bat coronavirus and an origin-unknown coronavirus could be considered [5].

The COVID-19 symptoms have reportedly ranged from mild to severe, which could lead to death. The prevalence of an asymptomatic form of this disease is yet to be determined. The affected patients presented with symptoms of severe pneumonia, including fever, fatigue, myalgia, dry cough, and dyspnea with respiratory distress [1]. Less common symptoms reported were rhinorrhea, diarrhea, headache, nausea, vomiting, and hemoptysis. Increasing evidence shows that coronaviruses are not always confined to the respiratory tract and that they may also invade the central nervous system, giving different neurological signs including acute cerebrovascular symptoms, impaired consciousness, anosmia, and ageusia [6,7,8]. More than half of patients with dyspnea needed flow oxygen therapy, non-invasive ventilation, invasive ventilation, and intensive care, and most of them worsened in a short period of time and died due to respiratory failure most often caused by acute respiratory distress syndrome (ARDS) and/or disseminated intravascular coagulation (DIC) [9,10,11,12]. High plasma levels of proinflammatory cytokines have been observed in individuals with severe disease, suggesting that a cytokine storm effect could be present, triggered by viral infection [10,11]. Several ongoing clinical trials are focusing on a combination of antiretroviral drugs, including lopinavir, ritonavir, rendesemir associated with monoclonal antibodies (tocilizumab), and chloroquine, associated with low-dose systematic corticosteroids [12,13,14]. Also, plasmapheresis has been found to be useful in the treatment of COVID-19 [15].

At present, the diagnosis of COVID-19 depends on real-time reverse transcriptase-polymerase chain reaction (RT-PCR), and it is assumed on the basis of symptoms of pneumonia. Chest computed tomography (CT) is recommended in suspected COVID-19 cases for assessment of disease extent and follow-up, as well as supplementing parts of the limitations of RT-PCR assays [16]. The findings most often reported on CT include ground-glass opacity (GGO) distributed in the lower lobes unilaterally or bilaterally, reticular and interlobular septal thickening, and GGO with consolidation and pure consolidation [8,17]. So far, only a few studies have reported radiological findings that are typical of embolism in COVID-19 patients [18,19]. We reported two clinical cases, both affected by COVID-19, showing different radiological features on CT scans, suggesting a possible different spectrum of action of SARS-CoV-2.

## 2. Case Presentations

### 2.1. Case 1

A 78-year-old woman was admitted to our Emergency Department due to the presence of fever, cough, fatigue, and dyspnea. The nasopharyngeal and oropharyngeal swabs were positive for SARS-CoV-2 infection using SARS-CoV-2 RT-PCR. Blood examination showed normal hemoglobin (Hb 13.1 g/dL), reduced mean cell volume (MCV, 80.5 fL), normal total white blood cell counts (5.82 × 10^3^/μL), and abnormal platelet counts (147,000/mm^3^). Increased values of C-reactive protein (15.28 mg/L) and D-dimer (255 ng/mL) were present. Troponin I was in the normal range (0.1 ng/mL). Other laboratory values, including electrolytes, creatinine, and liver enzymes, were normal.

**Figure 1:**
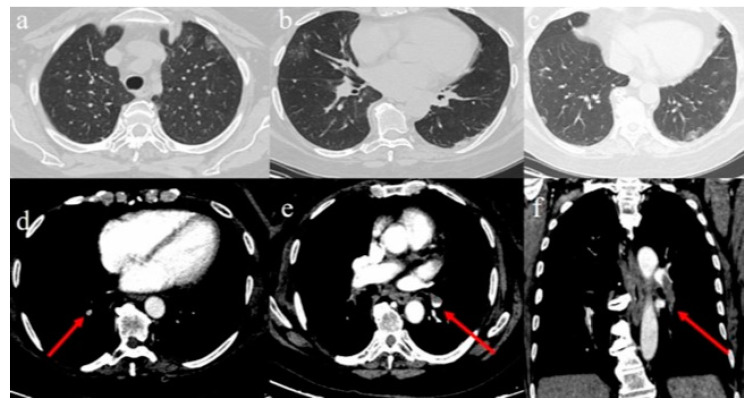
Chest computed tomography (CT) and CT angiography (CTA) of Case 1. Lung window (W: 1600 UH, L: −500 UH) of chest CT scan: findings of COVID-19 pneumonia with peripheral ground-glass opacities in the left superior lobe (**a**), in the middle lobe, with a posterior peripheral consolidation in the inferior left lobe (**b**); peripheral ground-glass opacities in the inferior lobes (**c**). Mediastinal window (W: 251 UH, L: 45 UH) of CTA scan: filling defect in a right subsegmental pulmonary artery branch for the inferior lobe (red arrow in (**d**)); an extensive filling defect in the lobar and in a segmentary pulmonary arterial branch for the inferior lobe (red arrow in (**e**) on the axial plane and in (**f**) on the coronal plane).

Arterial blood gas revealed a PaO_2_ of 79.0 mmHg, a PcO_2_ of 37 mmHg, and an SpO_2_ of 96%. No history of smoking was reported as well as any history of autoimmune, hematological, cancer, and thrombophilic diseases. The patient promptly underwent an unenhanced chest CT completed with chest CT angiography (CTA). The lung parenchyma, evaluated with high-resolution CT (HRCT) algorithm reconstruction, showed the presence of sporadic ground-glass opacities with subpleural distributions in the anterior left superior (Figure 1a) and in the posterior segments of right superior lobes and in the lateral segment of the middle lobe (Figure 1b). Ground glass opacities were also present in the inferior lobe with subpleural and posterior distributions, associated with an initial consolidation on the left side (Figure 1b,c). The CTA scan documented a minor embolism of some subsegmental branches for both inferior lobes (Figure 1d) and a filling defect involving the lobar and the segmentary branches of the left pulmonary artery of the inferior lobe, which is diagnostic for acute pulmonary embolism (APE) (Figure 1e,f). Based on these reports, treatment with lopinavir/ritonavir was started together with hydroxychloroquine and low molecular weight heparin. Thereafter, no other CT imaging was performed, and lower-limb compression ultrasonography performed after the first CT scan showed thrombosis in the right popliteal vein. Despite the increased dosage of anticoagulant therapy, the patient died a few days later.

### 2.2. Case 2

**Figure 2:**
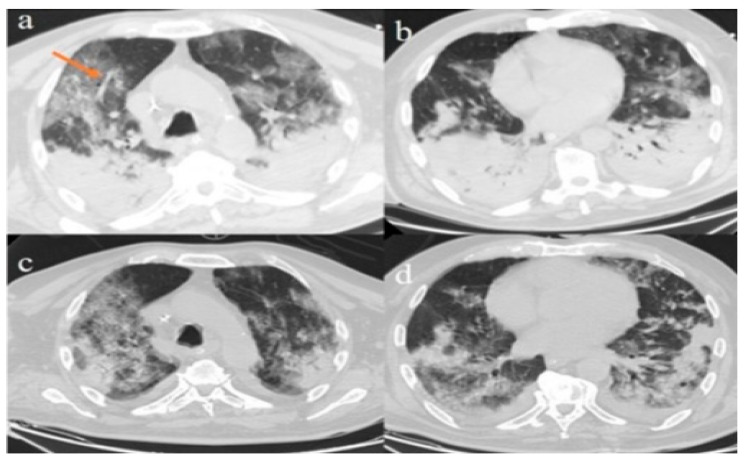
Chest CT of Case 2. Lung window (W: 1500 UH, L: −400 UH) of chest CT scan: ground-glass opacities in a crazy paving pattern with consolidations in the superior lobes with more posterior and peripheral distributions associated with severe consolidations in the inferior lobes (**a**,**b**) and vascular enlargement (orange arrow) (**a**); reduction of consolidations in the superior (**c**) and inferior lobes (**d**) after tocilizumab therapy.

A 72-year-old man was admitted to the Emergency Department due to the presence of acute dyspnea and fever. Nasopharyngeal and oropharyngeal swabs were positive for SARS-CoV-2 infection, and an interstitial lung involvement with initial alveolar consolidation was found on the chest X-ray. No history of smoking was reported. On admission, blood examination showed mild leukocytosis (10.640/mm^3^), abnormal platelet counts (70,000/mm^3^), a normal value of D-dimer (1.91 ng/mL), increased values of C-reactive protein (57.9 mg/L), and high level of glycemia (366 mg/dL). Other laboratory values, including the level of hemoglobin, electrolytes, creatinine, and liver enzymes, were in the normal range. Arterial blood exam revealed a PaO_2_ of 69.6 mmHg, a PCO_2_ of 63.2 mmHg, and SpO_2_ of 92.3%. The patient needed mechanical ventilation support in the intensive care unit. Therapy with lopinavir/ritonavir and hydroxychloroquine was started. Since there was evidence of clinical ARDS, we performed chest CT that showed bilateral diffuse ground glasses opacities with crazy paving patterns and consolidations in the superior lobes with peripheral and posterior distributions and severe consolidations of the inferior lobes (Figure 2a,b). Vascular thickness (Figure 2a) and pleural effusion were also described and present bilaterally. Due to the higher levels of interleukin (IL) 6 (1632 pg/mL), the patient started tocilizumab therapy. Chest CT performed 5 days later showed a mild radiological improvement with a reduction of consolidation areas in the superior and in the inferior lobes (Figure 2c,d). However, two days later, the patient died from ARDS complications.

## 3. Discussion

The typical hallmarks of COVID-19 pneumonia reported on CT scans are characterized by bilateral lung involvement, multifocal ground-glass opacities, and consolidation in a typical peripheral with a posterior-dependent gradient and more consolidation in the postero-basal regions [16,17,20]. Emerging atypical CT manifestations, including airway changes, pleural changes, fibrosis, and nodules, were also present in COVID-19 patients [8,16,17,20]. On imaging CT scans, the radiological finding of COVID-19 could be presented in different ways according to the patient’s age, immunity status, disease stage at the time of scanning, underlying diseases, and drug interventions. Lung APE is an emergent complication of COVID-19, and it was found only in a few studies [18,19,21]. However, APE’s occurrence in COVID-19 patients is still unclear. Some authors documented bilateral pulmonary embolism with negative lower-limb compression ultrasonography [18,19], whereas, more recently, a relation between COVID-19 disease and the risk of thrombotic or thromboembolic events, including venous thromboembolism, has been found [22]. In COVID-19 patients, abnormal coagulation parameters are usually associated with poor prognosis [9,10,23]. An acute infection as viral pneumonia could be associated with a transiently increased risk of venous thromboembolic events due to the epithelial damage and the platelet and endothelial cell dysfunctions that may contribute to the thrombosis [11,22]. However, so far, the site of initial infection with SARS-CoV-2 is still unknown, and the pathogenesis of COVID-19 is under investigation. Some studies revealed the presence of high levels of pro-inflammatory cytokines including IL-2, IL-7, IL-6, IL-10, granulocyte-colony stimulating factor (G-CSF), monocyte chemoattractant protein (MCP)-1 and macrophage inflammatory protein (MIP), and tumor necrosis factor (TNFα) in patients with COVID-19 [10,11,22,23]. This “cytokines storm” may have a major role in the pathogenesis of COVID-19 and could initiate viral sepsis and inflammatory-induced lung injury, leading to other complications including development of DIC related to a common coagulation activation and secondary hyperfibrinolysis condition, confirmed by elevated levels of fibrin-related markers (D-dimer and fibrin degradation products) [22,23]. On the other hand, in severe cases reported, COVID-19 can be complicated by ARDS, sepsis and septic shock, and multiorgan failure, including acute kidney injury and cardiac injury [24]. It’s known that ARDS can be derived from two possible pathogenic pathways [25]: a direct insult on lung cells (pulmonary ARDS (ARDS-p)) or indirectly (extrapulmonary ARDS (ARDS-exp)). In ARDS-p, the alveolar epithelium is primarily affected by producing a local alveolar inflammatory response while the capillary endothelium is roughly normal [25]. In ARDS-exp, the indirect insult primarily affects the vascular endothelium by inflammatory mediators through the bloodstream, leading to increased vascular permeability and interstitial edema. The inflammatory agents are more increased in the serum in ARDS-exp, while in ARDS-p, inflammatory agents are increased primarily in the bronco alveolar lavage (BAL). The radiological pattern in ARDS-p is characterized by a balance between ground-glass opacities and consolidations with a prevalence of alveolar consolidations in asymmetrical and non-dependent distribution; on the other hand, in the ARDS-exp, the typical pattern is characterized by a prevalent ground-glass opacities with a craniocaudally and sterno-vertebral gradient distribution [25]. In ARDS-exp, consolidation tended to be at middle and basal levels, also in vertebral position. We postulated that some radiological features found in our Case 2 could be compatible with radiological findings of both the ARDS-p and the ARDS-exp. Notably, it is possible to consider the co-existence of the two insults: one lung with direct injury (as pneumonia virus-induced) and with an indirect injury (through mediators related to cytokines activation stimulated by the presence of the virus) [25]. In COVID-19, dilatation and congestion of alveolar septal capillary and exudation of fluid in the alveolar cavity is seen in the early stage while the accumulation of a large number of cell-rich exudates in the alveolar cavity, vascular expansion, and exudation in the interstitium is seen in the rapid progressive stage. This mechanism could suggest a toxic cytokines mechanism mediated that could explain the feature of ARDS-exp. Despite the different radiological finding reported in Case 1, characterized by COVID-19 pneumonia and onset of APE, in this case, the gradient and the posterior distributions of GGO typical of COVID-19 pneumonia associated with the presence of minor embolisms could also be related to the toxic effect related to the “cytokine storms” seen in ARDS-exp [25,26]. These results highlighted the importance of an early combined therapy, including both anti-inflammatory and antiviral drugs associated with anticoagulant therapy [27]. Immunomodulant therapy, including plasmapheresis, could also be taken into account. Further autopsy or histological studies are needed to understand more pathological details of this disease.

## 4. Conclusions

In conclusion, special attention needs to be paid to the risk of developing APE in COVID-19 infection, and further laboratory investigation including D-dimer and coagulation exams associated with CTA and ultrasonography should be part of the assessments in COVID-19 patients in order to detect early signs of APE. It is also important to consider in COVID-19 patients with a clinical and radiological finding of ARDS both intra- and extrapulmonary mechanisms related to the “cytokine storms” in order to provide early use of a complete therapy, including antiviral, anticoagulant, anti-inflammatory and immunomodulant drugs.
